# Special Issue “Molecular Insight into Gestational Diabetes Mellitus”

**DOI:** 10.3390/ijms27031204

**Published:** 2026-01-25

**Authors:** Marcin Trojnar, Żaneta Kimber-Trojnar

**Affiliations:** 1 Chair and Department of Internal Diseases, Medical University of Lublin, 20-059 Lublin, Poland; 2 Chair and Department of Obstetrics and Perinatology, Medical University of Lublin, 20-090 Lublin, Poland

Gestational diabetes mellitus (GDM) is one of the most prevalent metabolic disorders complicating pregnancy worldwide, currently affecting up to 15–25% of pregnancies [1]. It is defined as glucose intolerance first recognized during gestation and has emerged as a major public health concern in parallel with the rising prevalence of obesity, advanced maternal age, and sedentary lifestyles [2]. Beyond its immediate obstetric implications—such as gestational hypertension, preeclampsia, macrosomia, neonatal hypoglycemia, and increased rates of cesarean delivery—GDM confers substantial long-term metabolic risks for both the mother and offspring, including an elevated likelihood of developing type 2 diabetes mellitus (T2DM), metabolic syndrome, and cardiovascular disease (CVD) later in life [3].

Physiologically, pregnancy is characterized by a progressive increase in insulin resistance, which ensures an adequate glucose supply to the growing fetus. Under healthy conditions, pancreatic β-cells adapt by expanding in mass and increasing insulin secretion, thereby maintaining euglycemia [4,5]. In GDM, however, this adaptive capacity is exceeded. Inadequate β-cell compensation in the face of increasing insulin resistance results in persistent maternal hyperglycemia, often accompanied by elevated circulating free fatty acids, dysregulated lipid metabolism, and excessive gestational weight gain [5,6]. These disturbances reinforce one another, creating a self-perpetuating cycle of metabolic stress.

A central feature of this dysmetabolic state is the emergence of chronic, low-grade, sterile inflammation. Hyperglycemia and lipotoxicity activate multiple inflammatory signaling pathways that impair insulin action in skeletal muscle and adipose tissue and simultaneously compromise pancreatic β-cell survival and function [5,7]. These interconnected inflammatory and metabolic networks—including nuclear factor kappa-light-chain enhancer of activated B cells (NF-κB), Toll-like receptors (TLRs), peroxisome proliferator-activated receptors (PPARs), sirtuins, phosphoinositide 3-kinase (PI3K), mammalian target of rapamycin (mTOR), AMP-activated protein kinase (AMPK), and NLRP3 inflammasome activation—form a mechanistic interface between metabolic overload, immune activation, and insulin resistance (contribution 1). Thus, GDM is increasingly understood not simply as a disorder of glucose metabolism but as a state of immunometabolic dysregulation.

This immunometabolic perturbation is particularly evident in the placenta, a transient yet highly specialized organ that integrates nutrient transport, endocrine signaling, and immune tolerance at the maternal–fetal interface. In GDM, the placenta exhibits characteristic structural and functional alterations, including increased placental mass, abnormal villous architecture, altered vascularization, and dysregulation of apoptotic and autophagic pathways [8]. At the molecular level, placentas from GDM pregnancies show enhanced expression of pro-inflammatory cytokines such as tumor necrosis factor-α (TNF-α), interleukin-1β (IL-1β), and interleukin-6 (IL-6), together with markers of oxidative stress and mitochondrial dysfunction. While controlled inflammatory signaling is indispensable for normal placental development, its chronic activation may impair placental efficiency, disrupt fetal nutrient delivery, and contribute to adverse metabolic programming in the offspring [9].

Metabolic programming extends beyond the intrauterine environment into the early postnatal period, where human milk represents a key biological conduit between maternal metabolism and infant development. Colostrum, produced during the first days after birth, is enriched in adipokines and hormone-like factors—including leptin, adiponectin, ghrelin, resistin, and insulin-like growth factor I (IGF-I)—that regulate appetite, energy balance, insulin sensitivity, and immune maturation (contribution 2). Maternal hyperglycemia and insulin resistance have been shown to alter the adipokine profile of colostrum, thereby potentially influencing neonatal satiety signaling, growth trajectories, and long-term susceptibility to obesity and metabolic disease [10].

Excessive gestational weight gain frequently coexists with GDM and represents an additional metabolic burden during pregnancy [11]. Defined as weight gain exceeding Institute of Medicine recommendations based on a pre-pregnancy body mass index, excessive gestational weight gain is independently associated with adverse maternal and neonatal outcomes and shares key pathophysiological features with GDM, including insulin resistance, chronic low-grade inflammation, and altered adipokine signaling [11,12]. These overlapping phenotypes highlight that GDM and excessive gestational weight gain are not isolated entities but rather interconnected manifestations of pregnancy-related metabolic dysregulation.

Within this framework, dipeptidyl peptidase-4 (DPP-4) has emerged as a potentially important molecular link between glucose metabolism, adiposity, and inflammation. Preliminary evidence indicates that circulating DPP-4 concentrations are elevated in women with GDM and in those with excessive gestational weight gain, correlating with indices of metabolic dysfunction (contribution 3). Although the precise role of DPP-4 in pregnancy remains to be fully elucidated, its established involvement in incretin degradation, immune modulation, and adipose tissue inflammation positions it as an intriguing biomarker and possible mechanistic contributor to gestational metabolic disorders.

These endocrine and enzymatic disturbances do not occur in isolation but intersect with host–microbial interactions during pregnancy. A growing body of evidence implicates the maternal gut microbiota as a critical regulator of metabolic homeostasis, with pregnancy-associated shifts in microbial composition linked to insulin resistance, inflammation, and altered energy balance [13]. Similar patterns of dysbiosis have been described in GDM, suggesting bidirectional interactions between maternal metabolism and the gut microbiome [14,15]. In this context, medical nutrition therapy represents a particularly attractive intervention which is capable of simultaneously improving glycemic control and favorably modulating microbial ecology [16].

Against this complex biological backdrop, the Special Issue Molecular Insight into Gestational Diabetes Mellitus brings together five complementary contributions that illuminate how immunometabolic, placental, endocrine, microbial, and biomarker-based pathways converge in GDM.

Zgutka et al. provide direct experimental evidence that placentas from GDM pregnancies adopt a distinctly pro-inflammatory phenotype driven by IL-1β- and Toll-like receptor-dependent signaling (contribution 1). Using placental tissue, explant cultures, and in vitro systems, the authors show that activating these pathways amplifies cytokine production via NF-κB-dependent mechanisms, reinforcing the concept that sterile inflammation is not merely a consequence but a central driver of placental dysfunction in GDM.

Extending the maternal–placental axis into early postnatal life, Lis-Kuberka et al. demonstrate that GDM is associated with selective alterations in colostral appetite-regulating adipokines, particularly ghrelin, in relation to disease severity and therapeutic approaches (contribution 2). These findings support the emerging view that maternal metabolic status during pregnancy leaves a biochemical imprint on human milk, thereby shaping early-life endocrine signaling and potentially influencing long-term metabolic programming in the offspring.

At the systemic level, Niebrzydowska-Tatus et al. identify elevated circulating DPP-4 concentrations in women with GDM and in those with excessive gestational weight gain, linking this enzyme to shared pathways of adiposity, dysglycemia, and lipid dysregulation (contribution 3). The associations between DPP-4, body composition, and metabolic indices highlight its potential utility as a biomarker that integrates multiple dimensions of gestational metabolic stress.

The role of the gut microbiota as a modulator of these processes is comprehensively addressed by Enache et al. (contribution 4). Their review underscores how pregnancy-associated dysbiosis may contribute to insulin resistance and inflammation while also emphasizing that medical nutrition therapy can beneficially reshape microbial composition and improve metabolic outcomes, positioning diet–microbiota interactions as a cornerstone of GDM management.

Finally, Inthavong et al. synthesize the current knowledge on a wide array of biochemical markers to predict and present a prognosis of GDM (contribution 5). Although no single biomarker can yet replace the oral glucose tolerance test, their analysis supports the concept that integrated, multi-marker strategies combining adipokines, inflammatory mediators, insulin resistance indices, and glycemic parameters may enable an earlier identification of at-risk women and more individualized clinical care.

Taken together, the contributions in this Special Issue portray GDM as a disorder of interconnected immunometabolic networks spanning the placenta, maternal circulation, the gut microbiota, and early postnatal endocrine signaling through human milk. By integrating molecular mechanisms with emerging biomarkers and modifiable environmental factors such as nutrition, this collection provides a framework for earlier prediction, targeted prevention, and more precise management of GDM in an era of increasing metabolic disease (Figure 1).

We invite readers to explore these contributions, which provide a comprehensive molecular perspective on GDM and highlight potential avenues for earlier prediction, targeted prevention, and improved management of this increasingly prevalent condition.

## Figures and Tables

**Figure 1:**
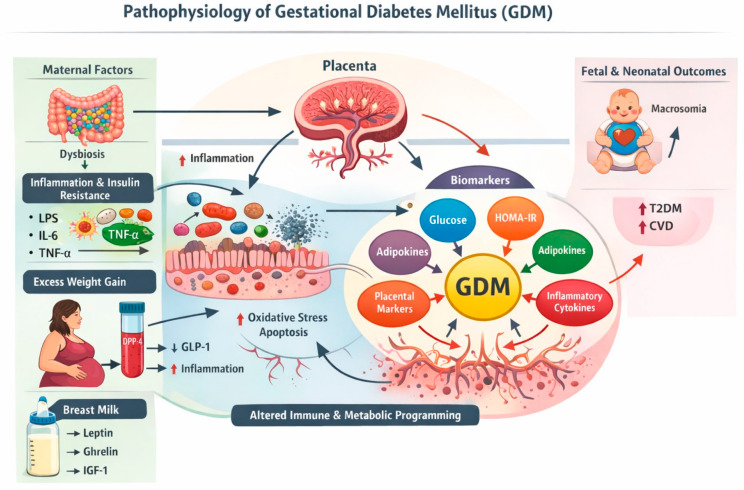
Schematic overview of the interconnected molecular, metabolic, and inflammatory mechanisms underlying gestational diabetes mellitus.

## Abbreviations

AMPK	AMP-activated protein kinase
CVD	cardiovascular disease
DPP-4	dipeptidyl peptidase-4
GDM	gestational diabetes mellitus
GLP-1	glucagon-like peptide-1
HOMA-IR	homeostasis model assessment of insulin resistance
IGF-I	insulin-like growth factor I
IL-1β	interleukin-1 beta
IL-6	interleukin-6
LPS	lipopolysaccharide
mTOR	mammalian target of rapamycin
NF-κB	nuclear factor kappa-light-chain-enhancer of activated B cells
NLRP3	NLR family pyrin domain containing 3
PI3K	phosphoinositide 3-kinase
PPARs	peroxisome proliferator-activated receptors
T2DM	type 2 diabetes mellitus
TLRs	Toll-like receptors
TNF-α	tumor necrosis factor alpha
